# Solution of the spatial neutral model yields new bounds on the Amazonian species richness

**DOI:** 10.1038/srep42415

**Published:** 2017-02-17

**Authors:** Yahav Shem-Tov, Matan Danino, Nadav M. Shnerb

**Affiliations:** 1 Department of Physics, Bar-Ilan University, Ramat-Gan IL52900, Israel

## Abstract

Neutral models, in which individual agents with equal fitness undergo a birth-death-mutation process, are very popular in population genetics and community ecology. Usually these models are applied to populations and communities with spatial structure, but the analytic results presented so far are limited to well-mixed or mainland-island scenarios. Here we combine analytic results and numerics to obtain an approximate solution for the species abundance distribution and the species richness for the neutral model on continuous landscape. We show how the regional diversity increases when the recruitment length decreases and the spatial segregation of species grows. Our results are supported by extensive numerical simulations and allow one to probe the numerically inaccessible regime of large-scale systems with extremely small mutation/speciation rates. Model predictions are compared with the findings of recent large-scale surveys of tropical trees across the Amazon basin, yielding new bounds for the species richness (between 13100 and 15000) and the number of singleton species (between 455 and 690).

Neutral dynamics, and the neutral models used to describe it, are one of the main conceptual frameworks in population biology and ecology^1^,^2^,^3^. A neutral community is a collection of different populations, such as different species (in ecological models) or different groups of individuals with identical genetic sequence (haplotypes, for example, in population genetics). All individuals undergo a stochastic birth-death process, where in most of the interesting cases the overall size of the community, *J*, is kept fixed or almost fixed. An offspring of an individual will be a member of its parent “group” (species, genotype) with probability 1−*ν*, and with probability *ν* it mutates or speciates, becoming the originator of a new taxon. A neutral process does not include selection: all populations are demographically equivalent, having the same rates of birth, death and mutations, and the only driver of population abundance variations is the stochastic birth-death process (also known as ecological/genetic drift or as demographic noise).

A neutral dynamics is relevant, of course, to any inherited feature that does not affect the phenotype of an individual, like polymorphism in the non-coding part of the DNA or silent mutations, but many believe that its scope is much wider. In particular, the neutral theory of molecular evolution^1^ and the neutral theory of biodiversity^2^ both suggest that even the phenotypic diversity observed in natural communities reflects an underlying neutral or almost-neutral process while the effect of selection is absent or very weak. Both theories have revolutionized the fields of population genetics and community dynamics, correspondingly, and (despite bitter disputes) their influence is overwhelming.

For a well-mixed (zero dimensional, panmictic) community the mathematical analysis of the neutral model is well-established, with the theory of coalescence dynamics^4^ and Ewens’s sampling formula^5^ at its core. However, the species abundance distribution (SAD) predicted by the well-mixed model, the Fisher log-series, fails to fit the observed statistics of trees in a plot inside a tropical forest. To overcome this difficulty, Stephen Hubbell suggested a simple spatial generalization of the neutral model, where a well mixed community on the mainland (a “metacommunity”) is connected to a relatively small island by migration, and immigrant statistics is given by Ewens’s sampling formula^2^,^6^. The abundance of a species on the island reflects, in this case, the balance between its mainland relative abundance (assumed to be fixed, as variations on the mainland are much slower) and local stochasticity. The resulting island statistics depend on two parameters only, the fundamental biodiversity number *θ* = *ηνJ*_*m*_ (*J*_*m*_ is the mainland abundance, *η* = 2 for a non-overlapping Wright-Fisher dynamics and *η* = 1 is for a Moran process with overlapping generations, see ref. 7) and *m*, the migration rate. This two-parameter model fits very nicely the SAD observed in local communities, and its mathematical simplicity allows for an exact solution in terms of zero-sum multinomials^6^,^8^; these two key ingredients contributed greatly to the success of Hubbell’s neutral theory^3^,^9^.

Still, this mainland-island model is only an approximation. The tropical forest plots used to validate it are not “islands” per se. Instead, they are arbitrary segments of very large forests on which censuses take place. Even the plot known as “Barro-Colorado Island (BCI)” is a 500 × 1000 m rectangle on an island whose area is 15.6 *km*^2^. In practice there is no natural distinction between the local population and its surroundings, they both are part of a continuous forest connected by local dispersal. In reality one should expect that the effect of the regional community on local populations (and vise versa) decreases with the distance from the edge, a phenomenon that has no analogous in Hubbell’s model.

Another motivation to extend Hubbell’s mainland-island model comes from recent large scale empirical surveys of tropical forests^10^. Fisher log-series appears to give the best fit for the observed SADs on different scales, suggesting an underlying neutral dynamics. However, when the results were extrapolated to the rare species regime, the value of Fisher’s alpha (which is equal to the parameter *θ* defined above, and corresponds to the number of singleton species, i.e., species represented by only one individual) turns out to be between 900 and 1200 (for the Amazon). On the other hand, to fit the SAD observed in the BCI plot to zero-sum multinomials the value *θ*~48 should be taken for the mainland community^8^. The huge difference between these two estimations is clearly related to the fact that only a small part of the Amazon basin acts as a regional pool for the BCI plot. However (See Supplementary section I for quantitative and qualitative discussion) for a well-mixed community the number of “local singletons” in every regional subcommunity (i.e., species who are represented by a single individual in this region) is the same as the overall number of singletons. The local Fisher’s alpha is much smaller than its global value only in models that allow for spatial clustering, like the model we present here.

Moreover, another Amazon basin study^11^ showed that range size of a species, even a hyperdominant one, is smaller than the basin itself. Even the most frequent species were found in 1/3−1/2 of the plots, and usually these plots cover a spatially compact region. To explain this phenomenon within the framework of the neutral theory (i.e., without spatial heterogeneity) one needs a spatially explicit model in which the species’ range size is limited by the spatial dynamics of individuals.

A solution for the generic problem of spatially explicit neutral dynamics is, for these reasons, greatly needed^12^,^13^. Several attempts have been made in this direction, both in the context of community ecology^3^,^14^,^15^,^16^,^17^,^18^ and in the context of population genetics^19^, but we believe that the novel solution presented here allows, for the first time, for a rigorous comparison between model and data for the numerically inaccessible regime, e.g., for community statistics of the whole Amazon basin.

## Methods

We consider a spatial system of *J* individuals, where in each elementary step one individual is removed at random (death) and is replaced by an offspring of another individual in its neighborhood (Moran process, *η* = 1). The recruitment kernel has width *σ*, i.e., the chance of the offspring of an individual at *r* to be recruited into a gap at *r*` is proportional to *exp*(−|*r* − *ŕ*|^2^/(2*σ*^2^)). Upon birth, the newborn takes the identity (species, haplotype etc.) of its mother with probability 1 − *ν* or mutates (speciates) and becomes the originator of a new taxon with probability *ν*. Recurrent mutations are not allowed, so every mutant is a singleton of a new type.

When *σ* → ∞ the system is well mixed and the results of the classical neutral model hold, meaning that the species abundance distribution is given by Fisher log-series


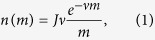


where *n(m*) is the average number of species represented by *m* individuals. Accordingly, the species richness (SR) in the community is given by


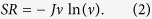


For finite *σ* the results must be different since the system is spatially correlated. Conspecific individuals are clumped and the chance that a dead individual is replaced by a newborn that belongs to the same species is higher. Accordingly, although the dynamics is still strictly neutral and is driven by fluctuations, the (per capita) effect of stochasticity on large populations is smaller than the effect on small populations, as the average number of intraspecific replacements is higher if the population is small. In section II of the Supplementary we show how this feature manifests itself in an effective description of species dynamics using a Fokker-Planck equation. The spatial structure enters this equation through the function *I(m*) that expresses the effective “interface area” of a species, or the chance (for a population of size *m*) of interspecific interaction in an elementary birth-death event. Once *I(m*) is known, one can find the SAD and, from its normalization condition, deduce the overall species richness.

## Results

The details of our analysis are given in Supplementary II. In two spatial dimensions and for small values of *ν* the mutation process does not affect the spatial structure of the community and one can implement the results obtained for the *ν* = 0 process^20^,^21^,^22^ to determine *I(m*). The resulting species abundance distribution is:


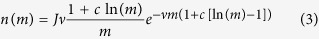


where *c* is a constant that depends (for small *ν*) only on *σ*^2^. *c* must decrease to zero when *σ* → ∞ in order to retrieve the Fisher Log-series in the well mixed limit. Our numerics fit excellently to (3), and the parameter *c* appears to satisfy *c* = [*a(σ*^2^ − 1) + *b*]^−1^, where *a* = 3.22 ± 0.03 and *b* = 2.58 ± 0.31 (see further discussion and numerical evidence in Supplementary IV). Note that in our process *σ* is always larger or equal to one, and that length is measured in units of 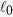, the typical distance between neighboring individuals. The overall species richness is given by the sum of *n(m*) over *m*, and (see derivation in the Supplementary) and may be approximated by,


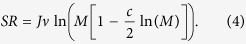


**Figure 1:**
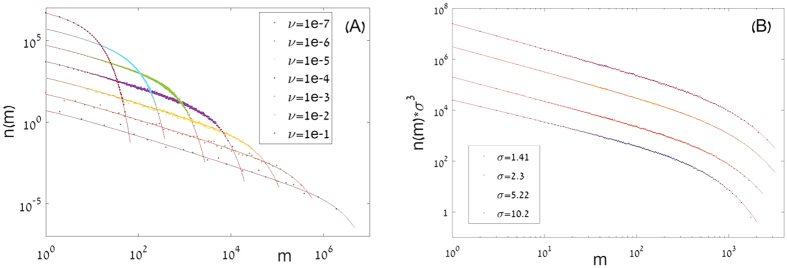
Species abundance distribution *n(m)* vs. *m, as* obtained from simulations of the spatially explicit neutral dynamics, is shown on a double logarithmic scale (Pueyo plots, as in ref. 36). Our numerical technique, using the backward in time approach suggested in ref. 18, is explained in Supplementary III. Results from a 5000 × 5000 square lattice (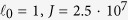, periodic boundary conditions) are depicted for different values of *ν* and *σ*. Numerical results for *n(m*) are represented by dots, full line is the theoretical prediction of Eq. (3). In panel (**A**) the results are shown for *σ* = 1 where *ν* varies between 10^−1^ and 10^−7^. In panel (B) the SAD is plotted, for the same system, now at fixed *ν* = 0.001 and different values of *σ*, between 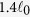 and 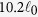. Datasets in panel (**B**) were shifted vertically by multiplying *n(m*)s by *σ*^3^. For all the SADs we implemented the logarithmic binning technique used in ref. 37.

**Figure 2:**
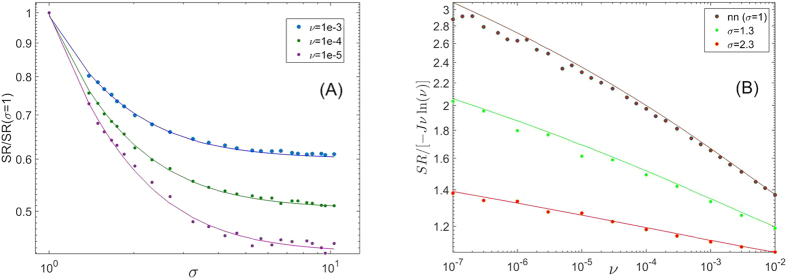
Species richness (SR) is plotted against *σ* for different values of *ν*: 10^−3^,10^−4^ and 10^−5^ [panel (A)]. Since the SR depends strongly on *ν*, we normalized the SR for each *ν* and *σ* by its *SR(ν,σ* = 1) (nearest neighbors) value. Circles are the results of numerical simulation (with the same 5000 × 5000 lattice used to obtain the results of Fig. 1), Full lines are the theoretical prediction, calculated from the sum over the SAD given in Eq. 3. In panel (**B**) we present the results for the species richness (to emphasize the deviation from the well-mixed prediction 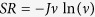, we divided the numerical results by this quantity). Each circle reflects a single run of the simulation, except the 4 circles of panel (B), *σ* = 2.3, *ν* < 10^−5^, that represent an average over five systems since the single system results were too noisy.

Here 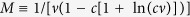 is, roughly speaking, the abundance scale above which the SAD starts to decay exponentially, so *M* sets the scale of the hyperdominant species. The deviation between the approximation (4) and the exact sum over *n(m*) is smaller than 5%. The excellent fit of these expressions to the results of large-scale numerical simulations is depicted in Figs 1 and 2.

As seen in Fig. 2A, the species richness in the community decreases as the recruitment length *σ* increases. Under neutral dynamics, small spatial patches undergo fixation by a single species, so when the system is divided into many patches with negligible inter-patch dispersal the number of species will be equal to the number of patches, much larger than the neutral theory prediction for a panmictic community (this result has been discussed recently in the context of “small island effect”^23^). As the recruitment length decreases the spatial distribution of species becomes less homogenous (clustered), with relatively smaller migration between different spatial locations, thus the overall species richness increases as well.

Another important quantity in a spatially structured community is the range size of a species. Under neutral dynamics, the range limit is determined by the combined effect of species age and dispersal range: all individuals of a species of abundance *m* are descendants of a single mutant who lived 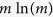 generations ago, and each birth is a random step of typical length *σ*. Accordingly, the geographic range size of a species of abundance *m* is *ξ*^2^(*m,σ*), where *ξ* is the typical length (measured in units of 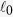). The value of *ξ* for the most abundant species is the correlation length of the system, since the community structure is uncorrelated beyond this length scale.

The spatial dynamics of a neutral species over generations is almost identical to branching-coalescing random walk^17^ or “Brownian bugs”^24^. Individuals tend to be clustered, as birth occurs only in the vicinity of another individual while death occurs everywhere. However, as long as spatial effects are considered, the “effective age” of a species is not 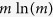 but *m*, since the extra logarithmic factor comes from intraspecific replacement processes that do not affect the spatial configuration. Accordingly, following^24^,^25^ one may expect that the species’ range size is,


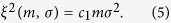


In Supplementary V this point is discussed and numerical evidence are shown to support 5, where *c*_1_ was found to be between 0.8 and 1.2.

Armed with these results, Eqs (3, 4, 5) we can now proceed to consider a problem which is otherwise inaccessible, both numerically and analytically: the predictions of the neutral model for a large scale spatial community and the relationships between these predictions and the outcomes of empirical surveys. We focus on the tropical tree community all across the Amazon basin, with about *J* = 4.3 × 10^11^ trees (free standing stems ≥ 10 cm diameter at breast height) spread over an area of 6.3 × 10^6^ km^2^ (meaning that 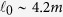)^11^. An attempt to simulate the neutral dynamics of such a system until equilibrium is clearly hopeless (see Supplementary III).

In ref. 11 one can find estimations of the SAD of frequent species and the length scales associated with their range. From these and similar results researchers have estimated the total number of tree species, the number of singletons and so on. As we mentioned above it was noticed^10^,^11^,^26^ that these empirical SADs are very close to the Fisher log-series, suggesting that the well-mixed version of the neutral model provides an appropriate description of this system. On the other hand the spatial structure of local communities, the finite range associated with different species, and the strong deviations from Fisher log-series in local communities like the BCI plot all show that the population is not panmictic (see ref. 12), meaning that deviations from the well-mixed neutral theory predictions are expected. Can one have the cake and eat it too? Our analysis provides a positive answer. To demonstrate that, we compare two features of the Amazonian trees community: the observed species richness and the range limit of the most abundant species.

In ref. 11, a sample of 553,949 trees (out of a forest of 4.3 × 10^11^ trees, sampling ratio *R* = 1.3 × 10^−6^) revealed *S* = 4962 different tree species. To be consistent with Eq. (3), the values of *σ* and *ν* should be calibrated. In particular, the SAD of a sample of *RJ* individuals, *n(s*), is related to the true SAD of a community with *J* individuals *n(m*) through (see ref. 7, Eqs 22 and 23),


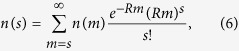


and a sum of *n(s*) over *s* yields the species richness *S(ν, σ*) in the sample.

Although the studies published so far do not provide exact numbers for the range size of different species, one may suggest a crude qualitative estimation. Of the six most abundant tree species listed in ref. 11, all with estimated abundance of 3−5 × 10^9^ trees, one species was found in about 50% of the surveyed plots (the maximum) and another is represented in only 7.4% of the plots (minimum). This provides, according to (5), a constraint on *σ*.

**Figure 3:**
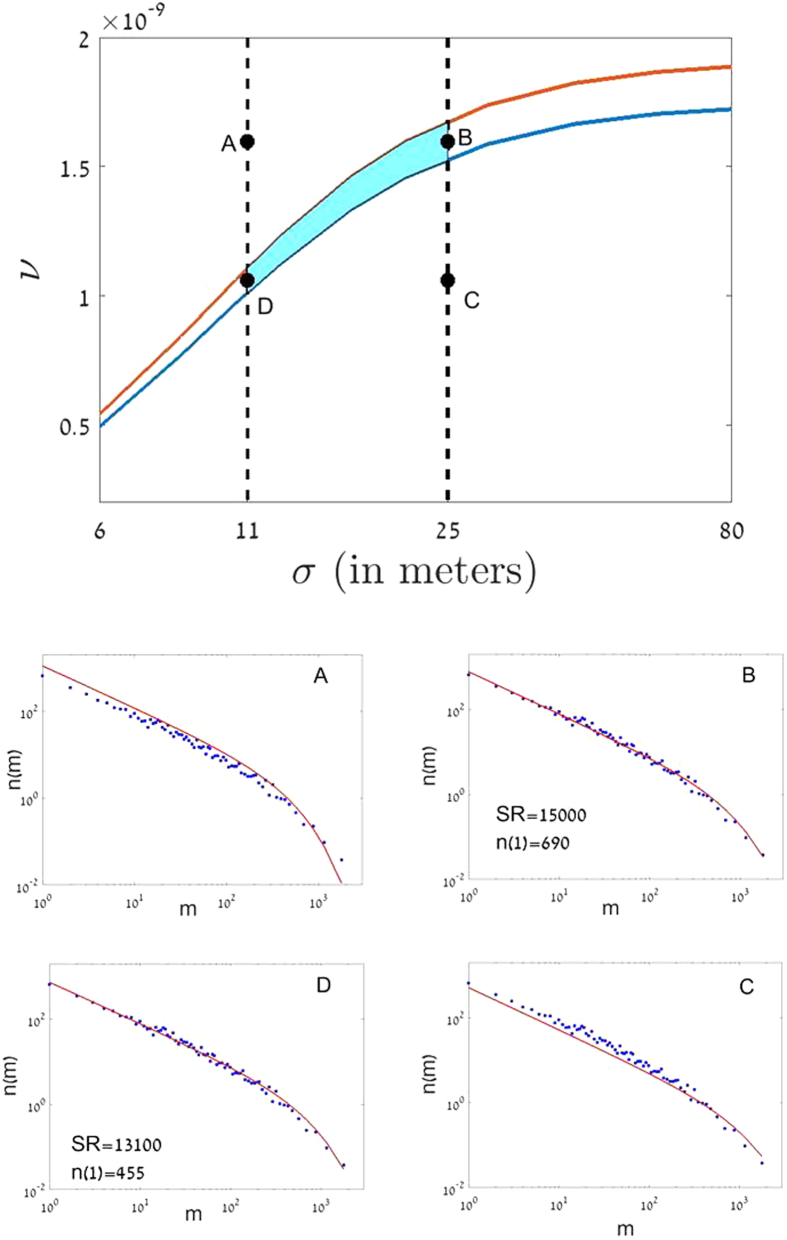
The spatial neutral theory predictions for the Amazonian tree flora. The abundance of 4962 tree species as measured in a sample of more than half a million trees in 1170 plots was reported by ref. 11. To be consistent with the spatial neutral theory presented here, *σ* and *ν* have to be chosen such that the species richness in the sample, *S(ν, σ*), is indeed 4962; this condition is fulfilled along the blue line in the *σ*-*ν* plane. As explained in Supplementary VI, the number 4962 is perhaps an underestimate, since the sampled individuals are spatially correlated, but the deviation is not larger than 8%, so we have plotted also the red line where *S(ν, σ*) = 5360. The vertical lines correspond to values of *σ* for which *ξ*^2^ is 7.4% of the Amazon basin for a species with abundance 3.7 × 10^9^, as reported for *Oenocarpus bataua*, and 48% for a species with abundance 5 × 10^9^, as reported for *Eschweilera coriacea*. The overlap between the two regions (shaded blue area) is the parameters regime in which both the species richness and the spatial deployment of species are consistent with the spatial neutral theory. In the lower panel we show the empirical SAD in the sample (circles) vs. the theoretical predictions of the spatial neutral theory (*n(s*) vs. *s* as obtained from Equation 6) for the points marked **A**, **B**, **C**, **D** in the upper panel, using a double logarithmic scale (Pueyo plot). One sees an excellent fit in the overlap regime and a very bad fit out of it.

In Fig. 3 we present the relevant region in the *ν*-*σ* plane: the intersection between the line on which *S(ν, σ*) yields the appropriate result and the *σ* regime that gives reasonable numbers for *ξ*^2^. Indeed, the SAD *n(s*) calculated for these parameters using (3) and (6) fits quite nicely the observed SAD obtained in the empirical survey. This implies that both the community structure (as reflected in the SR) and the spatial dynamics of individuals (recruitment kernel, for sessile species) may be explained by the neutral model for a certain range of parameters. The ability to bridge over the huge scale difference between the local processes, characterized by *σ* which is between 10−25 *m*, and the correlation length *ξ*, measured in thousands of kilometers, and the ability to make a connection between the community structure and the spatial structure of species, are both due to the analytic expressions (3–6).

Figure 3 shows that the region of parameters in the *ν*-*σ* plane for which the theory fits the empirical results is quite narrow, meaning that the predictions of the neutral theory are strong. The limitations on the possible values of *ν* and *σ* yield new estimations for the metacommunity species richness, (between 13,100 and 15,000) and for the number of singletons in the forest (Fisher’s alpha) (between 455 and 690). Note that these numbers are smaller than the estimates of ref. 11 (16,000 tree species, 730 singletons), which correspond to the *σ* → ∞ of our model.

## Discussion

The need to extend the neutral model and to consider a spatially explicit, continuous version of it, is not new. It has been emphasized in refs 3, 9, 12 and 13 and was the subject of a few previous works. Let us clarify the differences between these works and the new results presented above.

First, our expression for the SAD and the correlation length (Equations 3 and 5) disagrees with the scaling analysis suggested in ref. 14 (this scaling was already criticized in ref. 27, where it failed to fit numerical results). In particular, the correlation length scaling with *ν* is not a simple power law (see Equation (5)).

By the same token, the field theoretical analysis presented in ref. 16, and in particular the formula suggested for the species area curve (Eq. (10) of ref. 16) are in contrast with our simulation results and with Eqs (4 and 5), as these authors predict a purely linear dependence of the SR on *ν*.

In ref. 28, Fangliang He suggested a model that interpolates between the local community and the metacommunity versions of the neutral model. His model is, in fact, a mainland-island model, but He included speciation on the island and immigration/emmigration, so one can tune the parameters to imitate both meta (no migration) and local (strong migration, negligible speciation) communities. This approximation, also, neglects density correlations above the local community scale, or takes them into account in the migration parameters; it is difficult to see how to do that correctly to obtain 3, for example.

Muneepeerakul *et al*.^29^ have compared the SAD of a simulated two dimensional neutral model with a finite dispersal kernel with the corresponding SAD obtained from simulation of a river network (i.e., a collection of patches connected by directed links), finding an access number of low-abundance species in the river network topology. This insight is supported by the results we obtained for the one-dimensional version of the model considered here^30^, showing also a relatively large fraction

The analysis given here does not solve the species-area curve (SAC) problem. To do that one should provide a solution for the backward in time coalescence-death problem with inhomogeneous initial conditions, as pointed out by Etienne and Rosindell^13^. These authors have tried to extract the parameters *θ* and *m* for the mainland-island model from the parameters *ν* and *σ* of the spatially explicit model considered here, concluding that this mapping makes no intuitive sense and that, for realistic values of *ν*, the goodness-of-fit is low. A few steps towards an analytic solution for the backward in time coalescence problem were presented recently^31^,^32^,^33^,^34^,^35^, and we hope to address this question in future work.

The neutral model provides an extremely simple framework for the analysis of the dynamics of communities and populations on both ecologic and evolutionary time scales. The analysis presented here allows, for the first time, for a reliable comparison between empirical and theoretical results for a spatially structured, large metacommunity, and provides a connection between the local scale and the global scale. An increase of the number of surveyed trees in the Amazon basin by an order of magnitude, say, is a formidable challenge; On the other hand, a set of local measurements that will allow for an estimation of the interface function *I(m*), or the use of genetic markers to retrieve the recruitment kernel *σ* both seem to be within reach and, as we showed here, will allow one to examine the predictions of the neutral theory in the metacommunity level.

## Additional Information

**How to cite this article:** Shem-Tov, Y. *et al*. Solution of the spatial neutral model yields new bounds on the Amazonian species richness. *Sci. Rep.* **7**, 42415; doi: 10.1038/srep42415 (2017).

**Publisher's note:** Springer Nature remains neutral with regard to jurisdictional claims in published maps and institutional affiliations.

## Supplementary Material

Supplementary Information
