# Supplementary material for: Solution of the spatial neutral model yields new bounds on the Amazonian species richness
Source: Sci Rep. 2017 Feb 17;7:42415. doi: 10.1038/srep42415 (PMC5314346; doi:10.1038/srep42415)
Supplement: Supplementary Information [file srep42415-s1.pdf]

Supplementary information for:
Solution of the spatial neutral model yields new bounds on the Amazonian species richness

Yahav Shem-Tov, Matan Danino and Nadav M. Shnerb
Department of Physics, Bar-Ilan University, Ramat-Gan IL52900, Israel

I. NUMBER OF SINGLETONS IN A SUBCOMMUNITY

In this section we would like to present some quantitative and qualitative arguments explaining the factors that determine the number of observed singletons in a local or regional community.

Let us begin, first, with a well mixed community of J individuals for which the SAD is a Fisher log-series. One samples a region of this community which contains, say RJ individuals with $R < 1$. If there are $n(1)$ singletons in the whole forest, how many "local singletons" $\tilde{s}$ one will find in a fraction R of the forest, i.e., how many species will be represented by only a single individual in the subcommunity?

The answer is quite surprising,

$$\tilde{s} = n(1).$$

To see that, let us think about $n(1)$, the mean number of species that have exactly one individual in the whole forest J (real singletons). A fraction R of these singletons will be found in the sample, so the contribution of real singletons to $\tilde{s}$ is $Rn(1)$.

Now let us consider $n(2)$, the doubletons. If one samples each individual with probability R, there is a chance that one individual of a certain doubleton will be sampled while the other one is out of the sample, then this species will be a singleton in the subcommunity. The chance for that is clearly $2R(1 - R)$. Similar contributions for $\tilde{s}$ are $3R(1 - R)^2 n(3)$ and so on. Overall, the number of singletons in the subcommunity will be

$$\tilde{s} = Rn(1) + 2R(1 - R)n(2) + 3R(1 - R)^2 n(3)... = \sum_{m=1}^{J} mR(1 - R)^{m-1} n(m). \tag{S1}$$

If the metacommunity SAD is a Fisher log-series and if ν is small then $n(m) \approx n(1)/m$, and the sum in Eq. (S1) gives $\tilde{s} = n(1)$. The loss of real singletons (species for which $n(m) = 1$) in the sampling process is compensated exactly by the chance to sample only one individual from doubletons or tripletons or in general m-tons, yielding the same absolute number of singletons as long as the number of individuals in the samples is large, i.e., if $RJ >> 1$ (clearly if only one individual is sampled the number of singletons decreases; for the result of Eq. (S1) to hold one must be able to neglect correlations in the sample).

In a system with spatial structure the situation is different. To understand that qualitatively, let us consider a "maximally compact forest", where every species is localized within a square or a circle where no other species is present. In such a case an area that contains RJ individuals will have $\tilde{s} \sim Rn(1)$: almost all the local singletons will be real singletons of the whole forest, since the chance to have only one member of an m-ton within the area and the $m - 1$ others out of the area is negligibly small. These two extreme scenarios give the general insight: as the forest becomes more compact (the lower σ is), the number of local singletons s for a fixed area (fraction R of the forest) decreases.

Given that, one can see that the value $\theta \sim 48$, assumed for the mainland in order to fit the zero-sum multinomials to the Barro-Colorado plot data [S1] is incompatible with the number of singletons, of order 1000, obtained in [S2], *if* the mainland community is panmictic. In fact, the situation is even worse: since the two estimations differ by a factor of 20, it implies that even in the compact forest case, where the number of local singletons is minimal, one have to assume that 1/20 of the amazon basin, an area of about $300,000 km^2$, acts as an effective "mainland" (regional pool) for the local community in the BCI plot. These considerations highlight the need for the spatially explicit neutral model presented here, in which the regional SAD changes gradually as a function of length scale.

II. DERIVATION OF EQS. (3-5):

Technically speaking, the neutral dynamics is a "technicolor" version of the well known voter model [S3]. In the original voter model any individual has one of two colors, or opinions, and in an elementary timestep an agent is chosen at random to change its color, accepting instead the color of one of its (randomly chosen) neighbors. In many works one considers only the four nearest neighbors sites as the "neighborhood", this corresponds to the $\sigma = 1$ limit of our model.

A system under voter dynamics ends, inevitably, with a fixation of the population by one color (like any Markov process with an absorbing state). A neutral game proceeds according to the same rules, with the exception that the agent accepts its neighbor's opinion with probability $1 - \nu$ and, with probability ν, becomes the originator of a new color (note that, unlike the two allele model considered by [S4], in the infinite allele case considered here recurrent mutations are not allowed and a brand new species appears in every mutation).

Like the traditional voter model, the neutral dynamics may be analyzed using a "backward in time" (coalescence) approach, becoming a coalescence random walk ($A+A \to A$) process [S5]: every individual selects its parent from its neighborhood and coalesces with it. The resulting genealogic tree (for a $1d$ example with nearest neighbors dynamics) is illustrated in Fig S1a, where red lines representing ancestral relationships merge until the dynamics reaches the most recent common ancestor (MRCA). Mutation/speciation events are represented by short black lines that cut the genealogical lines, and all the leaves connected to a certain mutant by lines without mutations carry the same color, i.e, they belong to the same species. This property of the model facilitates the simulation of a neutral dynamics [S6], and we have implemented the backward in time strategy to simulate the process as explained in Supplementary III.

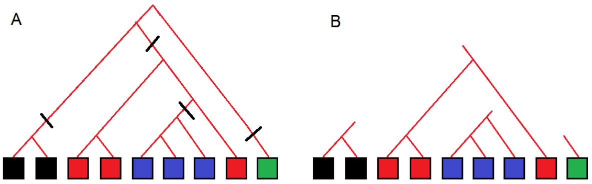

FIG. S1: Two cartoons showing a possible genealogy under neutral dynamics with $\sigma = 1$. In (A) the full genealogy of a $1d$ coalescence process with 9 individuals is presented. Every mutation (represented by thick dark lines cutting the line) generates a new species, and conspecific individuals at present have the same color. Panel (B) shows the same genealogy when simulated only until the last relevant mutation: coalescence history and mutations in the "missing" part of the tree are irrelevant.

While implementing a backward in time approach for the numerics, our analytic arguments are based on the forward in time evolution of the system and focus on the interface area. To begin, let us consider (as in Figure S1) the neutral dynamics in $1d$ (a model considered by geneticist, see [S7]). Looking at a species represented at present by m individuals (e.g., for the red species in Fig. S1 $m = 3$, for the black $m = 2$), one realizes that its dynamics is governed by two processes: weak losses at a rate νm per generation due to mutations, and an unbiased diffusion in abundance space associated with the birth-death dynamics. Clearly, the strength of diffusion for m is proportional to $\tilde{I}$, the *interface* area between color segments. For example, the red species in Fig. S1 has four interfaces, while for the blue one $\tilde{I} = 2$. Assuming a narrow distribution of the number of interfaces around an average $\tilde{I}(m)$ (our simulations suggest a Poisson distribution), one may write a Fokker-Planck equation for the single species abundance dynamics,

$$\frac{\partial P(m,t)}{\partial t} = \frac{\partial^2 [I(m)P(m,t)]}{\partial m^2} + \nu \frac{\partial [mP(m,t)]}{\partial m} \tag{S2}$$

where $P(m,t)$ is the probability of a certain species to be represented by m individuals at t (t is measured in generations). $I(m)$ is the chance of an interspecific interaction in an elementary birth-death event; for example, under nearest-neighbor dynamics on a square lattice $I(m) = \tilde{I}(m)/4$ in two spatial dimensions. Once $I(m)$ is known, one may solve (S2) for $\dot{P} = 0$ and the equilibrium species abundance distribution (SAD) is given (up to normalization) by,

$$P_{eq}(m) = \frac{e^{-\nu \int^m dx \frac{x}{I(x)}}}{I(m)}. \tag{S3}$$

This formula is valid in any dimension and for any recruitment kernel, but $I(m)$ depends on both. Now we would like to suggest an expression for $I(m)$ in two dimensions.

Two is the critical dimension of the coalescing random walk problem [S5, S8] and of the first passage time in general [S9], with logarithmic corrections to the mean field (well-mixed) results. Under a simple voter-model dynamics without mutations, the chance of the lineage of an individual to survive after t generations (backward in time) goes like $ln(t)/t$ (as opposed to $1/t$ above $2d$) [S8]. Accordingly, to keep the population fixed the average number of offspring of a surviving individual after t generations has to be $t/ln(t)$. Therefore, up to logarithmic corrections, the age of a species with abundance m is

$$t(m) \sim m \ln(m).$$

The neutral dynamics without mutation (i.e., the forward in time voter model) satisfies Eq. (S2) with $\nu = 0$. A simple scaling argument shows that to have $t \sim m \ln(m)$,

$$I_{2d}(m) = \frac{m}{1 + c \, \ln(m)}. \tag{S4}$$

Since $I(m)/m$ measures the "effective interface area" of an individual (i.e., its chance to be replaced by an individual of a different species), it must converge to one when $\sigma \to \infty$ (in the well mixed system, assuming $J >> 1/\nu$, all species fractions are of zero measure and the effect of intraspecific replacements is negligible, meaning that the interface area of every individual is one). In $2d$ and for small values of ν the value of c depend only on σ^2, since the spatial deployment of particles in a diffusion limited coalescence process depends only on this parameter. In section IV of this Supplementary we show a perfect fit to $c = [3.22(\sigma^2 - 1) + 2.85]^{-1}$. Plugging this expression into Eq.(S3) the SAD is found to be the one given in Eq. (3) of the main text.

The next step is the calculation of the overall species richness. Eq. (3) is normalized such that the overall number of individuals in the community, given by $\sum_m mn(m)$, is equal to J. The species richness is then given by $SR = \sum_m n(m)$. Since the argument of the exponent in (3) decays faster than exponentially when $m > M$, where $M \equiv 1/[\nu(1 - c[1 + \ln(c\nu)])]$, one may replace the sum by an integration over $1/I(m)$ from one to M, yielding Eq. (5) of the main text.

III: SIMULATION TECHNIQUE

The simulation of neutral dynamics involves an individual based process with birth, death and mutation/speciation. In its simplest (forward in time) form, one starts with a system of J individuals. During each elementary timestep one individual (say, a tree) is chosen at random to die and another individual is chosen, again at random, to give birth to an offspring that will fill the gap. With probability $1 - \nu$ this offspring carries the identity of its mother (it belongs to the same species/haplotype) and with probability ν it mutates/speciates and becomes the originator of a new taxon.

In a spatially explicit model the individual that give birth to the offspring is chosen at random from the spatial neighborhood of the dead individual, meaning that the distance between the mother and its offspring is limited. We used a Gaussian recruitment kernel. The dead individual is chosen, again, at random. If its location is $\mathbf{r}$, then the chance that the mother of the offspring that fills the gap is at $\mathbf{r}'$ is given by,

$$P(r') = \frac{1}{2\pi\sigma^2} e^{|\mathbf{r}-\mathbf{r}'|/2\sigma^2}. \tag{S5}$$

To implement this kernel on a two dimensional square lattice, we have adopted the following procedure. If the location of the dead tree is $[k, \ell]$, we pick a random number from a uniform distribution between zero and 2π to determine an angle θ, and another random number ρ from a normal distribution of width σ' to determine the distance. The mother of the newly recruited offspring is then chosen to be at $[k + round(\rho cos\theta), \ell + round(\rho sin\theta)]$, where "round" is the function that rounds each number to the nearest integer. If both $round(\rho cos\theta) = 0$ and $round(\rho sin\theta) = 0$, two other values for ρ and θ are picked until the reproducing tree is chosen.

The effective value of σ, i.e., the characteristic length of the recruitment kernel, is larger than σ' since the distance between the offspring and its mother is at least one lattice site. To calculate σ given σ' we have simulated a two dimensional random walk on a lattice using the same procedure and measured the variance (over many histories) of its location as a function of time. This variance is growing linearly, and its slope is defined as σ^2, as demonstrated in Figure S2. When the recruited offspring is taken only from the nearest neighbors of the dead tree, $\sigma = 1$, and this is the minimal value of σ.

This forward in time simulation of the neutral process is simple, but it is very difficult to make sure that the system is indeed at equilibrium. The time to the most recent common ancestor (T_{MRCA}) for the neutral dynamics with J individuals is J generations (in a $2d$ spatial system it T_{MRCA} is slightly larger, still the order of magnitude is similar), so to make sure that the system have forgotten its initial conditions one would like to preform about J^2 elementary steps (J steps for each generation) and for systems larger than $J = 10^6$ this task becomes impossible.

To overcome this difficulty we have adopted the backward in time (coalescence) technique suggested in [S6]. Instead of killing a tree and than replacing it by an offspring, we start with the system at present (without specifying the species of different trees), generate the genealogy with mutations and recover the species affiliation from it. In each elementary timestep an individual is chosen at random, chooses the location of its mother according to the recruitment kernel, and jumps to this lattice site. If this site is occupied, the mother and the offspring coalesce to a single individual. If the destination site is empty (meaning that the only descendents of the mother at the destination site who are represented in the community at present are those of this specific offspring) the offspring moves to the destination point.

This backward in time dynamics generates the full genealogy of all the individuals until the MRCA. This, however, is still too much for our numerics, since the coalescence times for a few random walkers on a two dimensional lattice

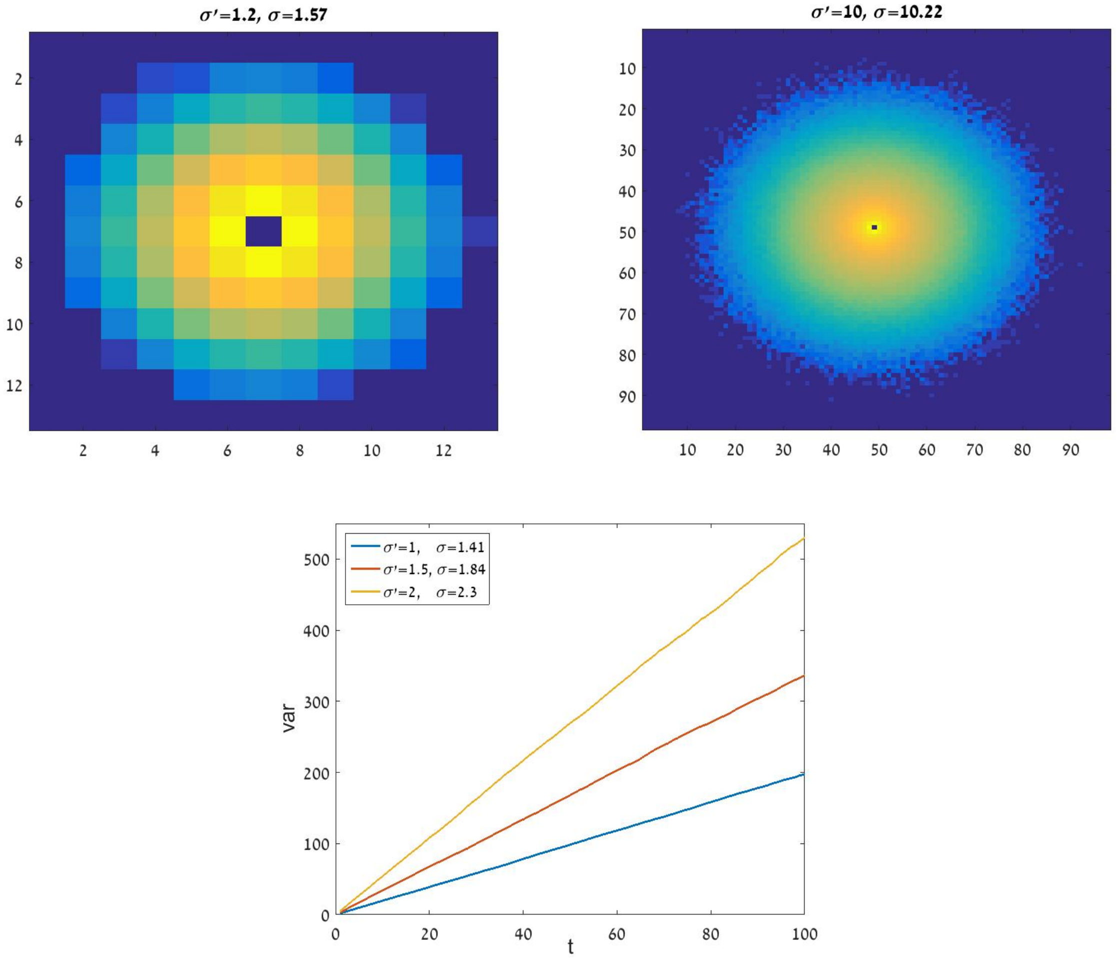

FIG. S2: The spatial dynamics of the numerics. The neutral process takes place on a square lattice, with a single tree in each site. One tree is chosen at random to die, then one of its neighbors is chosen to produce an offspring that will replace it. The chance of a neighbor at a distance r to reproduce is given by the procedure described in the text: first choosing its vertical and horizontal coordinates from the Gaussian kernel with width σ', than rounding these numbers to get two integers, and then, if both numbers are zero, picking another set of coordinates until getting a nonzero distance. The results of this process are depicted in the two upper panels, where the site of the dead tree is in the center (dark blue) and the (logarithm of the) chance of the reproducing tree to be at a certain site is given by the color of this site, ranging from yellow (high chance) to blue (low), for two different values of σ. In the lower panel we show the variance of 100000 runs (of 100 timesteps each) versus time. The slope of this curve determines the effective width of the recruitment kernel σ, as described in the text.

are also very long. However, we do not need to simulate the system to the MRCA because of the mutations. When mutation occurs in the forward in time process a new species emerges and from now on all the individuals that belong to this species have a "memory" only up to their originator. Accordingly, when an individual is chosen in the backward in time simulation, with probability $1-\nu$ it jumps to a new site and with probability ν it disappears from the system, meaning that this individual is the originator of a new taxon, and all its descendent at $t=0$ (present) are identified and "colored" (actually, numbered) with a unique color that correspond to this species/taxon. The whole procedure is exemplified (in $1d$) in Figure S1 above. The number of generations needed until the MRCA of the most ancient species is of order $1/\nu$, and since the number of individuals is decreasing (backward in time) due to the coalescence and the mutation, the number of elementary timesteps is much smaller and we can simulate systems of up to 10^8 individuals for values of ν down to 10^{-7}.

IV: DETERMINING THE VALUE OF $C(\sigma)$

As mentioned above [Eq. (S4)], the "effective interface area", $I(m)$ of a species with m individuals is $m/[1{+}c\ \ln(m)]$. Clearly, c must be a decreasing function of σ^2, approaching zero as $\sigma \to \infty$ to yield the well mixed result for the SAD, i.e., the Fisher log-series.

To find c, the value of $I(m)$ was monitored numerically using simulations of 5000×5000 systems with different values of σ and ν. $I(m)$ was calculated directly by checking, for every of the m individuals of a species, its chance to be replaced by an individual from other species using the weighted recruitment kernel described in Figure S2 above. We then plotted $m/I(m)$ as a function of $\ln(m)$ (figure S3, left) and calculated the slope of this curve using linear regression. The fit yields the value of c and its confidence intervals. Using these results, we plotted the values of $1/c$ vs. σ^2 (Figure S3, right. Error bars cannot be seen since the confidence intervals are very small) to obtain the dependence of c on σ, namely:

$$c = \frac{1}{a(\sigma^2 - 1) + b}, \tag{S6}$$

where $a = 3.22 \pm 0.03$ and $b = 2.58 \pm 0.31$.

The resulting values of c were found to be almost independent of ν (since the spatial structure is ν independent for small ν).

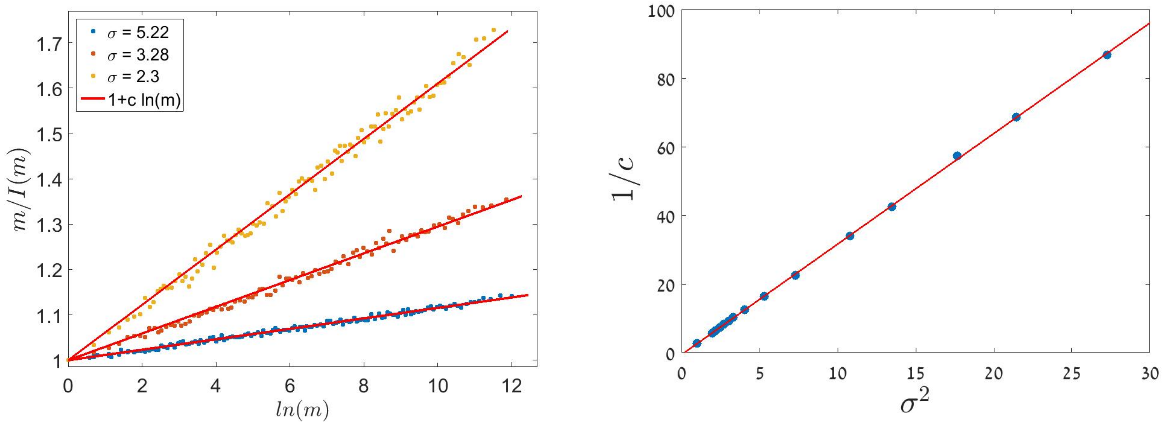

FIG. S3: Left panel: A plot of $m/I(m)$ vs. $\log(m)$ for different values of σ. The points are the results obtained from a numerical simulations with $\nu = 10^{-5}$ (binned, the actual results are very noisy, of course) and the full lines represent fits to $1 + c\ln(m)$ with different values of c. Right panel: $1/c(\sigma)$, as measured from the $I(m)$ graphs like those considered in the left panel, is plotted against σ^2 (simulations with $\nu = 10^{-5}$, $\nu = 10^{-4}$ and $\nu = 10^{-3}$ yield the same numbers). The fit to a straight line suggest the relationship $c = [3.22(\sigma^2 - 1) + 2.58]^{-1}$.

V: THE RANGE SIZE ξ^2

One of the main quantities considered through this paper is the spatial range associated with a species of abundance m. Generally speaking, the spatial range may be defined as the area where one can find individuals of the specific species, and we named this quantity $\xi^2(\sigma, m)$, where the length scale ξ is known as the correlation length [S10].

The definition of a range size for a species, in particular when one would like to compare it with empirical evidence, is an ambiguous task [S11]. One possibility is to define the range according to the maximum linear extent, i.e., the maximum distance between two conspecific individuals r_1, in such a case ξ is defined as $r_1/2$. This definition, based on extreme values, seems to yield an overestimate for the number of the plots in which the species is observed. Another possibility is to use the radius of gyration r_2 [S12], which is the square root of the average squared distance between each pair of individuals. Since the radius of gyration of a uniform disk of radius r is $r/\sqrt{2}$, we define ξ as $\sqrt{2}r_2$. If the distribution of individuals in the plane is nonuniform and populations tend to cluster close to their center of mass, r_2 underestimates the species range size.

As explained in the main text, our prediction (based on the theory of reproducing random walkers, or Brownian bugs) is $\xi = c_1 \sigma \sqrt{m}$ for a species of abundance m. Figure S4 demonstrate this feature for the two measures above - radius of gyration and maximal linear extent - for different values of σ. Indeed, $\xi/(\sigma\sqrt{m})$ is independent of both σ and m and is very close to unity, slightly above when the maximal linear extent is considered and slightly below when the radius of gyration is used to estimate ξ.

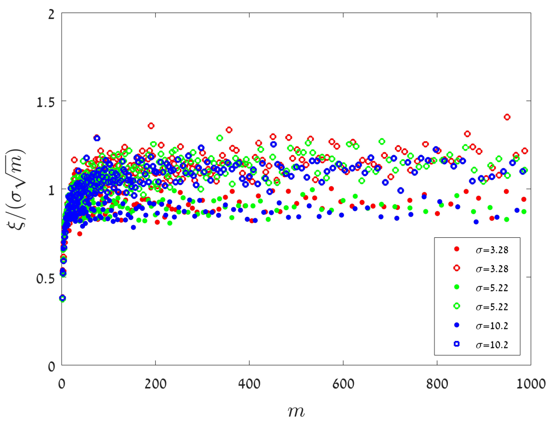

FIG. S4: The correlation length ξ, in units of $\sigma\sqrt{m}$, is plotted against m. The open circles correspond to a calculation of ξ from the maximum linear extent $r_1(m)$, where $\xi = r_1/2$. The filled circles correspond to the same quantity, but now ξ is defined as $\sqrt{2}r_2$, where r_2 is the radius of gyration. Clearly $\xi/(\sigma\sqrt{m})$ is independent of m for large m, and the quantity $\xi/(\sigma\sqrt{m})$ is σ independent. Results were obtained from a numerical simulation of the neutral model, 5000×5000 sites with $\nu = 10^{-4}$.

VI: DATA ANALYSIS

The empirical datasets analyzed here were published in the paper of Ter Steege et. al. [S13] three years ago. Total number of 553,949 trees ($dbh > 10$cm) were identified in 1170 plots in the amazon basin, and the SAD of this sample is given in the Supplementary of this paper (Appendix S1, column C).

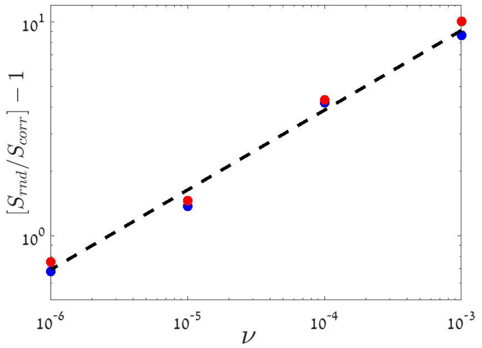

FIG. S5: The ratio $Z = (S_{rnd}/S_{corr}) - 1$ vs. ν for $W = 90000$, $k = 30$ (blue circles) and $W = 40000$, $k = 20$ (red), both plotted on a double logarithmic scale. One observes that the inefficiency ratio decreases exponentially with $\ln(\nu)$, suggesting the dependency $\ln(Z) = 4.89 + 0.38\ln(\nu)$ (dashed line). Extrapolating this dependency to $\nu = 2\ 10^{-9}$ (which is bigger than the upper limit for ν) one finds $Z = 0.065$. In figure 3 of the main text we used $Z = 8\%$, to be on the safe side.

Ter Steege et. al. have estimated the abundance of each tree species in their list, and used this estimation (fig. 2 in their paper) to find the value of θ, assuming that the SAD is indeed a Fisher log-series. However, it is well known that trees in the tropical forest are clustered, if only because the finiteness of the recruitment kernel. This implies that if one individual of a species was found in a plot, the chance to find another individual from the same species in this plot increases, so the trees in the census are not a random sample of the forest population.

To overcome this difficulty we have decided to stick to a more robust quantity: the species richness in the sample. In an uncorrelated sample one expects that the species richness is given by an integral of $n(s)$ (as given in Eq. (6) of the main text) over s. When the sampling itself is spatially correlated the chance of finding an individual that belongs to an already detected species is higher, so the observed species richness is lower.

In figure S5 we present the results of numerical experiments. After simulating a neutral system until it reaches its MRCA as described above, we have sampled W individuals at random for a few times and calculated the average species richness in the sample S_{rnd}, then we sample individuals from W/k^2 different $k \times k$ squares all over the system to get S_{corr}. The ratio $Z = (S_{rnd}/S_{corr}) - 1$ is then plotted against ν, and the results are extrapolated to the relevant values of ν.

[S1] R. S. Etienne, Ecology letters **8**, 253 (2005).
[S2] J. F. Slik, V. Arroyo-Rodríguez, S.-I. Aiba, P. Alvarez-Loayza, L. F. Alves, P. Ashton, P. Balvanera, M. L. Bastian, P. J. Bellingham, E. van den Berg, et al., Proceedings of the National Academy of Sciences **112**, 7472 (2015).
[S3] T. M. Liggett, *Stochastic interacting systems: contact, voter and exclusion processes*, vol. 324 (Springer Science & Business Media, 2013).
[S4] K. Korolev, M. Avlund, O. Hallatschek, and D. R. Nelson, Reviews of modern physics **82**, 1691 (2010).
[S5] D. Ben-Avraham and S. Havlin, *Diffusion and reactions in fractals and disordered systems* (Cambridge University Press, 2000).
[S6] J. Rosindell and S. J. Cornell, Ecology Letters **10**, 586 (2007).
[S7] J. F. Wilkins and J. Wakeley, Genetics **161**, 873 (2002).
[S8] P. Krapivsky, Physical Review A **45**, 1067 (1992).
[S9] S. Redner, *A guide to first-passage processes* (Cambridge University Press, 2001).
[S10] S. Azaele, A. Maritan, S. J. Cornell, S. Suweis, J. R. Banavar, D. Gabriel, and W. E. Kunin, Methods in Ecology and Evolution **6**, 324 (2015).
[S11] K. J. Gaston, Trends in Ecology & Evolution **11**, 197 (1996).
[S12] J. A. Bissonette, *Wildlife and landscape ecology: effects of pattern and scale* (Springer Science & Business Media, 2012).
[S13] H. Ter Steege, N. C. Pitman, D. Sabatier, C. Baraloto, R. P. Salomão, J. E. Guevara, O. L. Phillips, C. V. Castilho, W. E. Magnusson, J.-F. Molino, et al., Science **342**, 1243092 (2013).
